# Designing and Validation of Health-Related Quality of Life Inventory for Family Caregivers of Hemodialysis Patients

**DOI:** 10.30476/IJCBNM.2020.83081.1118

**Published:** 2020-04

**Authors:** Seyedeh Azam Sajadi, Abbas Ebadi, Seyed Tayeb Moradian, Roghayeh Akbari

**Affiliations:** 1 Department of Nursing Management, School of Nursing , Aja University of Medical Sciences, Tehran, Iran; 2 Behavioral Sciences Research Center, Life style institute, Baqiyatallah University of Medical Sciences, Tehran, Iran; 3 Atherosclerosis Research Center, Baqiyatallah University of Medical Sciences, Tehran, Iran; 4 Department of Nephrology, School of Medicine, Health Research Institute, Babol University of Medical Sciences, Babol, Iran

**Keywords:** Dialysis, Family caregivers, Inventory, Psychometric, Quality of life, Questionnaire

## Abstract

**Background:**

Family caregivers are important sources of care for hemodialysis patients. Although caring for a family member is a pleasant feeling, experiencing lots of physical and psychological caregiving burden influences the quality of life among family caregivers of hemodialysis patients. This study aimed to design and validate the quality of life inventory for family caregivers of patients on hemodialysis.

**Methods:**

A sequential-exploratory mixed method was conducted in Tehran, Iran, in 2017-2018. In the qualitative phase, the researcher conducted in-depth semi-structured interviews with 19 participants. Finally, a pool of 93 items was extracted from this phase. Then, psychometric properties such as face validity (Impact Score>1.5), content validity ratio (CVR>0.63), content validity index (Item Content Validity Index: ICVI>0.78 , Scale Content Validity Index/Average: SCVI/Ave>0.8) and Kappa value (Kappa>0.7, internal consistency (Cronbach’s alpha>0.7), relative reliability (ICC: interclass correlation coefficient), absolute reliability (Standard Error of Measurement: SEM and Minimal Detectable Changes: MDC), convergent validity (Correlation Coefficient between 0.4-0.7), interpretability, responsiveness, feasibility, and ceiling and floor effects were assesse.

**Results:**

The quality of life inventory for family caregivers of hemodialysis patients was developed with 34 items and five factors (namely patient care burden, conflict, positive perception of situations, self-actualization, fear, and concern). The findings confirm that the scale is acceptable regarding validity, reliability and other measurement features.

**Conclusions:**

This inventory is consistent with the health care status in Iran. Therefore, it can be used to measure the quality of life among family caregivers of hemodialysis patients.

## INTRODUCTION

One of the consequences of the enhanced life expectancy is the high prevalence of chronic diseases. ^1^ Chronic kidney disease, with an impact on 5%-10% of the world’s population, has been raised as a global public health concern in the world. ^2^ The prevalence and incidence of chronic kidney disease are also significantly increasing in Iran in recent years. ^3^ The patients with chronic diseases are mostly taken care at home by family members. ^4^ Studies show that the family caregivers play a critical role in managing the disease, caring for, and increasing the quality of life in patients with chronic renal failure and those undertaking hemodialysis. ^5 , 6^ In Asian countries, due to the stable and traditional family structure, the families of patients with dialysis take responsibility for the treatment of these patients. ^6^ In Iran, individuals are also deeply committed to traditions, and there are strong emotional relationships among family members. This traditional structure is one of the leading support sources for the patients. ^7^ Caregivers of hemodialysis patients face a large number of problems due to their frequent visits to the hospitals, the use of multiple drugs, and improper diets. They prefer the patient’s needs to their own needs and ultimately devote less time to perform health promotion behaviors, which in turn has adverse effects on their health and quality of life. ^8^ One of the major indices of health and well-being is the quality of life.2 Several definitions have been proposed in various sciences for the term “quality of life.” The World Health Organization defines the quality of life as an individual’s understanding of one’s status in life and in the cultural context and value systems, where they are living by their goals, expectations, standards, and concerns. ^9^ Since this concept is not directly measurable and is of a mental nature, the use of a valid and reliable tool is of paramount importance to properly understand it. ^10^ Patients undergoing hemodialysis experience a different life pattern compared to many other patients with chronic diseases. In such a life pattern, the dependence on the dialysis machine is an integral part, which also influences their caregivers’ quality of life. Therefore, specific local tools can provide a better understanding and ultimately a more comprehensive assessment of the caregiver’s status to adopt effective interventions to improve the quality of life among these individuals. Accordingly, the researcher aimed to bridge this gap in the literature and carry out the present study to design and validate the Health-Related Quality of Life (HRQOL) Inventory for Family Caregivers of Hemodialysis Patients.”

## MATERIALS AND METHODS

A sequential exploratory mixed research was conducted in qualitative and quantitative phases in Tehran, Iran, in 2017-2018. 

### *Qualitative Phase*

Since the quality of life is a context-based construct, ^9^ as noted in the introduction section, hemodialysis patients have special conditions compared to other chronic patients. These special conditions, including dependence on the dialysis machine, lead to changes in the lives of their family caregivers. Thus, in the first phase of the study, a qualitative method was used to understand the construct of the quality of life and its dimensions in family caregivers of patients undergoing hemodialysis. Among the different methods of qualitative studies, qualitative content analysis is one of the best methods for analyzing qualitative data in different areas of nursing and can provide insight into complex interactions. It can also be used in the designing or developing the stages of a measuring instrument. ^11^ Therefore, in this phase, qualitative content analysis method with a conventional approach was employed. In-depth and semi-structured interviews were conducted with 19 participants selected by purposive sampling. Maximum variability of the samples selection was considered from social and demographic characteristics. Key participants of this study were those over 18 years of age who were members of the patient’s family, had at least 6 months of caregiving experience, had the most involvement in patient care, had rich experiences and were capable of talking about the research question. The exclusion criterion was refusal to continue participation. The participants were personally informed about the details of the study, and confidentiality of any disclosed information. Family caregivers were assured that withdrawal from the study had no effect on the process of providing care to their patients. Written informed consent was obtained from all the participants. At the beginning of each interview, they were assured of the confidentiality of their information.

### *Quantitative Phase*

In the second phase of the study, a descriptive cross-sectional study was conducted to examine the psychometric properties. 

### *Face and Content Validity*

In order to determine the content validity, 12 family caregivers and 12 experts were asked to provide their comments on the inventory. The subjects were selected using conventional sampling. In this stage, the items were examined regarding face validity (Impact Score>1.5), content validity ratio based on Ayre and Scally’s ^12^ table (CVR>0.63), content validity index (Item Content Validity Index: ICVI>0.78 & Scale Content Validity Index/Average: SCVI/Ave>0.8), and Kappa value (Kappa>0.7). Finally, the final decisions were made by the indices mentioned above and comments collected from the research team on the deletion, modification, and inclusion of the items. To calculate the Scale Content Validity Index (SCVI), we first calculated the ICVI value for each item in the inventory, and then the mean of total ICVI was calculated for all items. ^13^

### *Item Analysis*

In the item analysis phase, a preliminary study was conducted on 50 main participants before running factor analysis to initially assess the adequacy of the number of items and identify defective items. The subjects were selected by conventional sampling. In this stage, if the correlation coefficient between the item and the whole inventory was smaller than 0.3, the item was removed. If the coefficient of correlation between tow items was higher than 0.7, one of those terms was also eliminated. Additionally, if the reliability level was reduced with the removal of an item, it showed that this item played a useful role in coordination with other items; thus, the item was appropriate. ^13^

### *Construct Validity*

Factor analysis is one of the best methods used to assess the construct validity. ^14^ According to the rule of thumb, the sample size of 300 cases is generally considered good for factor analysis. Therefore in this phase, 300 family caregivers who met the inclusion criteria (age>18 years, being a family member and having a patient care experience) were selected through convenient sampling and exploratory factor analysis, maximum likelihood method, and Varimax rotation was used to extract the latent factors. The maximum variability of the samples was considered from social and demographic characteristics. For statistical analysis, SPSS software, version 24, was used. The labeling was done based on the logic of alignment of the items and the theoretical background we obtained from the qualitative phase.

### *Convergent Validity*

For assessment of the convergent validity, the tool must be compatible with other tools measuring the same construct. ^13^ In this research, to assess the convergent validity, 300 participants through convenient sampling, by considering the maximum variety of samples, were asked to simultaneously complete the final version of the researcher-made inventory and QOL Short Form (SF-8) Instrument. The SF-8 is a shortened version of SF-36. The 36-Iitem short form health survey questionnaire (SF-36) is a widely used, standard instrument for evaluating health-related quality of life. ^15^ SF-8 was found to be useful, practical, and less time-consuming instrument in assessing health-related quality of life compared to the SF-36. The brevity of SF-8 has made it an ideal tool to assess the health-related quality of life, especially in large observational studies. ^16^ SF-8 measures eight dimensions of health, namely vitality, physical functioning, bodily pain, general health perceptions, physical role functioning, emotional role functioning, social role functioning, and mental health. However, our inventory had 34 questions and 5 dimensions to explain the quality of life of family caregivers with hemodialysis patients. In the three dimensions (patient care burden, conflict, fear and concern) of our tool, the impact of care on physical, psychological, individual and social dimensions of life was considered. This is in line with the dimensions of SF8. In addition to those two dimensions (positive perception of situations, self-actualization), it deals specifically with the positive effects of caring on the quality of life among family caregivers.

### *Reliability*

To determine the reliability of the inventory, internal consistency, relative and absolute reliability were assessed. To measure the relative reliability, we assessed the interclass correlation coefficient (ICC), and the Standard Error of Measurement (SEM) and Minimal Detectable Changes (MDC) were calculated to measure absolute reliability. Internal consistency refers to the correlation between the items in a tool. For measuring the internal consistency, Cronbach’s alpha was assessed. Cronbach’s alpha was calculated by completing the inventory by 3013 family caregivers. The alpha value should be 0.7 or higher for an item to remain in an instrument. ^17^

In this study, the inventory was completed by 30 family caregivers ^13^ Given the maximum variety of samples through convenient sampling at two different time intervals (within two weeks) in the same samples, ICC analysis was performed for the scores obtained from the two tests. Also, the correlation coefficient was assumed to be higher than 0.8, which indicates acceptable reliability. ^13^ The following equation was used to calculate the standard error of measurement:

SEM=SD√1–ICC 

SD is the standard deviation of the sum values obtained in test and retest phases, and the ICC is the coefficient of repeatability. To calculate the MDC, we used the following equation:

MDC=SEM×z×√2

The MDC can be calculated as a percentage of the “MDC%” to determine the actual relative changes after treatment or between repeated measurements over time to further show the relative value of the random error of measurement.

MDC%=(MDC÷mean)×100

“MDC%” is acceptable if it is smaller than 30%, and the excellent MDC% value is assumed to be below 10% ^13 , 18^

### *Responsiveness*

Regarding responsiveness, we expect the construction to be able to show the status of deterioration or improvement for individuals within a given period. ^19^ One of the methods to determine responsiveness is hypothesis testing. ^19^ In this research, this method was used to determine the responsiveness of the developed scale. For this purpose, the inventory was filled out by two groups of family caregivers (n=300) with different situations (based on income status: adequate, moderate and inadequate income) and the differences between the three groups was determined, using ANOVA and the LSD test (Post hoc analysis). 

### *Interpretability*

Interpretability refers to the qualitative significance of “Minimal Important Changes” in an instrument score. According to the COSMIN (COnsensus-based Standards for the selection of health Measurement INstruments) checklist, the benchmarks involved in the scope of the interpretability are calculating MIC, determining ceiling and floor effects, describing the distribution of total scores in samples, and determining the percentage of the missing items and the adequacy of the sample size. ^20^ To calculate the Minimal Important Change, the standard deviation of variations between the test-retest should be multiplied by the average effect size of 0.5. ^21^ The MIC must be higher than the MDC. ^22^ The ceiling effect occurs when the majority of the respondents choose the upper limit of a scale, and the floor effect occurs when the majority of the participants choose responses that are at the lower limit of the scale. ^23^ This index should be below 20%. ^24^ In this study, the ceiling and floor effects were also calculated as percentages for the total score of the instrument and the score obtained for all subscales to assess the discrimination power of the instrument and the distribution of responses. Another method to verify interpretability is to examine the distribution of scores in the samples. For example, the mean and standard deviation of the response is expected to vary in different groups. Accordingly, the average quality of life for different classes of participants in the present study was calculated based on the developed instrument. The other method used to confirm the interpretability of the dimensions is to calculate the frequency of non-responded (missing) items. It is desirable if the value ranges between 15-20%. ^25^ One of the ways to control the missing data is to replace them with the mean score. ^26^ This alternative method was used. In this research, however, attempts were made to minimize the missing items through asking for the family caregivers’ contribution and providing them with the instrument at the right time.

### *Feasibility*

Feasibility or ease of use is defined as the easy retrieval and practicality of an instrument in measuring the concerned construct. ^11^ In this study, the frequency of the responses and the frequency of non-responded items were determined for each item, and an accurate factor analysis was run to avoid a lengthy inventory.

### *Scoring Items*

The current inventory consists of a 5-point Likert scale (Strongly disagree, Disagree, Neither disagree nor agree, Agree and Strongly Agree). The inventory scores range from zero to 100. For this purpose, the following scores are transformed to standard scores through using the linear scoring. The higher an individual’s score is, the higher his/her quality of life will be.

$\mathrm{Transformed Score}=\frac{\left(\mathrm{Actual raw score}-\mathrm{Lowest possible raw score}\right)}{\left(\mathrm{Highest possible raw score}-\mathrm{Lowest possible raw score}\right)}\times 100$

Following the two qualitative and quantitative (psychometric) phases, the final version of the inventory with 34 items was developed.

This research has been approved by the Baqiyatallah University of Medical Sciences, Ethics Committee and has a code of ethics (ethical code:IR. BMSU.REC.1395.38). Since the most prominent point in conducting any research is to respect the patients’ dignity and human rights, the moral considerations of the Helsinki declaration and Committee on Publication Ethics (COPE) were observed by the researchers in this study. 

## RESULTS

### *Qualitative Phase*

Interviews with 19 participants led to the formation of 1637 codes, 25 subcategories, 11 category and 3 themes. These themes included post-traumatic growth, chronic care subjective burden, and objective chronic care burden. Finally, in this phase, a pool of 93 items was extracted (first draft of the inventory).

### *Quantitative Phase*

*etermining Validity*

At the end of this stage, according to the calculations performed for the face and content validity, the number of pool items decreased from 93 to 39 for reasons such as overlap or poor validity. In the present study, the mean value of content validity index was 89% for the whole inventory. The researchers usually assume the numerical value of 0.9 as perfect and the numerical value of 0.8 as the lower limit to confirm the content validity of a scale. ^13^

### *Item Analysis*

At this stage, Cronbach’s alpha was estimated to be 0.888. The correlation coefficient between two items was less than 0.7 in all cases. In this phase, the number of items was reduced from 39 to 36 items. The removed items are as follows:

a. Taking care of the patient has caused a reduction in my pride and selfishness.
b. Caring for a dialysis patient made me more responsible
c. I am satisfied with my performance as a care-giver.

### *Factor Analysis*

In this research, the quality of life construct, which consisted of 36 items, was evaluated regarding construct validity using two methods of factor analysis and convergent validity. To form the clusters, exploratory factor analysis, and Varimax rotation test was performed. The Kaser-Meyer-Olkin (KMO) value was calculated to determine the adequacy of the sample size and the Bartlett test for sphericity was used to check the item correlation matrix (Table 1).

**Table 1:** KMO sampling adequacy index and Bartlett test results

Kaiser-Meyer-Olkin Measure of Sampling Adequacy		0.890
Bartlett’s Test of Sphericity	Approx. Chi-Square	4618.445
	df	630
	Sig	P<0.001

**Figure 1:**
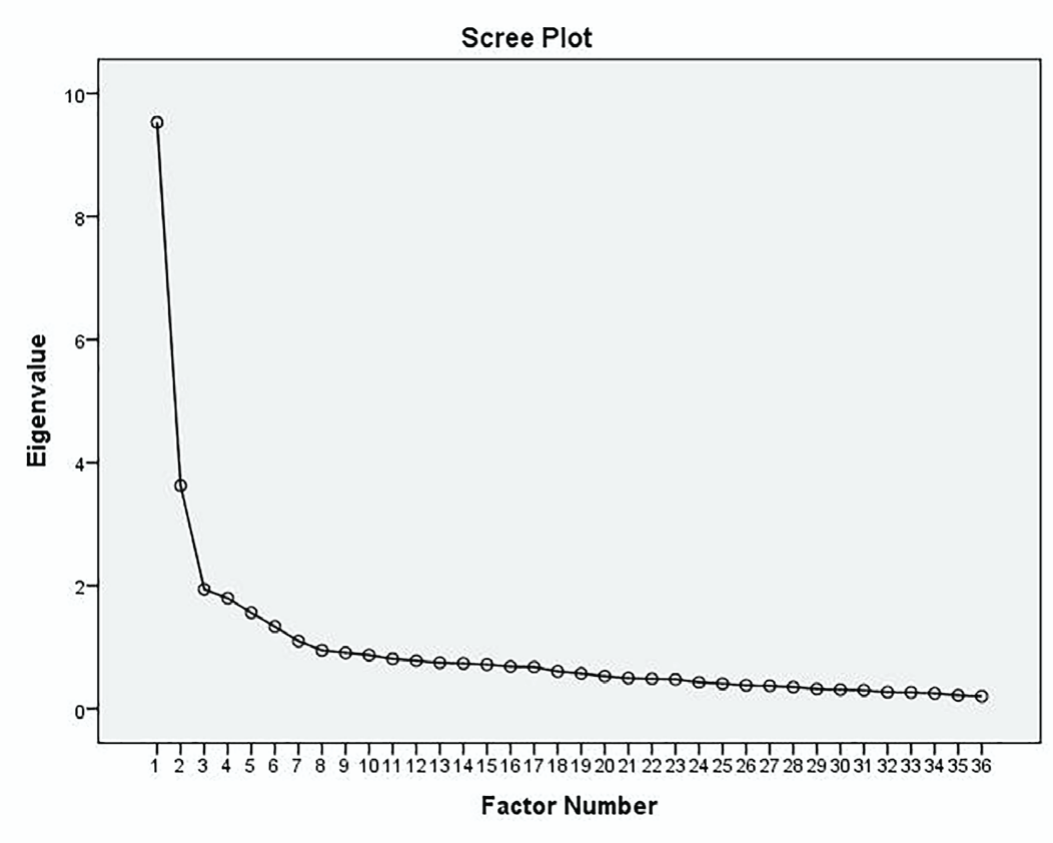
Determining the Number of Factors Constructing the Quality of Life Inventory for Family Caregivers of Hemodialysis Patients

In the next step, the latent factors in the test were extracted using likelihood method and Varimax orthogonal rotation under the assumption of independence for the factors. In this model, seven factors were extracted according to eigenvalues greater than 1 and scree plot (Figure 1). Finally, the 5-factor model had a better fit regarding the logic of alignment and labeling. The five latent factors accounted for eigenvalues of 9.53, 3.62, 1.94, 1.79, and 1.56, respectively. These five extracted factors explained 46% of the total variance of variables of this inventory. 

The factors extracted from the factor analysis using the Varimax rotation and the factor loading are presented in Table 2.

**Table 2:** Labeling each factor based on the relevant items by factor loading

No	Items	Factor loading				
Factor I	Factor II	Factor III	Factor IV	Factor V
1	Taking care of the patient has caused me physical pain.	0.738				
2	Dialysis patient care limits my decision about the future.	0.734				
3	Taking care of the patient has made me downgrade my health.	0.721				
4	I often feel physically exhausted.	0.711				
5	I mentally feel tired because of dialysis patient care.	0.708				
6	Patient care has made me forget my interests and preferences.	0.695				
7	Patient care has prevented me from exercising or having beneficial physical mobility.	0.693				
8	Dialysis patient care has disrupted my routine life.	0.668	0.354			
9	My daily life has lots of fluctuations due to unstable conditions of my patient.	0.660				
10	As my family member became sick, my need for medical and pharmaceutical treatment was further enhanced.	0.618				
11	My efficiency at work or home has been reduced.	0.611				
12	I am afraid of the indefinite future I have for the patient care.	0.586				
13	I feel less focused.	0.572				0.461
14	I am not satisfied with the quality of my sleep.	0.560				
15	I feel depressed for taking care of the patient.	0.500	0.431			
16	I am often worried.	0.471				0.413
17	I feel being ignored.	0.426	0.423			
18	I am worried about my financial status.	0.420				
19	I feel valued by others because of taking care of the patient.*					
20	I want to assign taking care of my patient to another person.		0.566			
21	As a result of the dialysis patient care, I feel the house is psychologically disturbing.	0.389	0.555			
22	I am embarrassed for my patients’ behaviors, which are due to kidney failure.		0.546	0.316		
23	I am worried about others’ judgment about how my patient care works.		0.496			
24	It seems that others have inappropriate care expectations from me.	0.398	0.414			
25	I am afraid that the way I take care of my patient harms him/her.		0.393			0.386
26	I hold a favorable view towards life because of taking care of a dialysis patient.			0.776		
27	Patient care made me more patient against problems.			0.507	0.473	
28	I am satisfied with my social relationships.			0.486		
29	Caring for a patient undergoing dialysis brings me comfort.			0.449	0.426	
30	I am satisfied with my life as a caregiver.*					
31	Patient care has empowered my sense of humanity.				0.868	
32	Patient care has strengthened my spirituality.				0.801	
33	I feel better about taking care of my patient.			0.525	0.534	
34	I am worried about the complications of dialysis and the improper functioning of the dialysis machines.					0.571
35	I am afraid that my patient’s condition is getting worse.			0.302		0.486
36	I suffer from my patients’ suffering imposed by illness and dialysis.					0.454

Factor I: Patient care burden, Factor II: Conflict, Factor III: Positive perception of conditions, Factor IV: Self-actualization, Factor V: Fear and concern.

* No factor load on any of the factors.

There was an attempt to transfer the cross-loaded items based on factor loadings to those with higher loads, but the items that had high factor loadings in the two factors (>0.4) were considered in addition to the scientific logic of the item and the factor. This process, of course, needs to be examined later in the confirmatory factor analysis. Accordingly, the item “Patient care made me more patient in confrontation to problems” was transferred from Factor III to Factor IV. Also, the other two items “I feel less focused” and “I am often worried” were transferred from Factor I to Factor V because of higher consistency. The item “I feel being ignored” with a similar factor loading for Factor I and Factor II was assigned to Factor II due to higher consistency. 

The two items “I feel valued by others because of taking care of the patient” and “I am satisfied with my life as a caregiver” were excluded from the inventory since they were loaded under none of the factors. Hence, the number of items in the inventory was reduced from 36 to 34 items. Subsequently, each factor was labeled according to its items. These factors included patient care burden, conflict, and positive perception of situations, self-actualization, fear and concern.

### *Determining Reliability*

As shown in the Table 3, the Cronbach alpha values obtained for each factor and the whole inventory were desirable.

**Table 3:** Cronbach’s Alpha coefficient by dimensions and the whole inventory

Factor	Lable	No. of items	Cronbach’s alpha Coefficient
1	Patient care burden	15	0.93
2	Conflict	6	0.66
3	Positive perception of situations	3	0.66
4	Self-actualization	4	0.87
5	Fear and concern	6	0.82
.	Total	34	0.93

The value obtained for the interclass correlation coefficient was close to one, and the lower bound of the confidence interval was also high for each of the factors as well as for the whole scale, indicating the acceptable reliability of the proposed tool. These results and those of a survey on the absolute reliability of Quality of Life Inventory for Family Caregivers of hemodialysis patients are presented in Table 4. 

**Table 4:** The Absolute Reliability of Quality of Life Inventory for Family Caregivers of Hemodialysis Patients

Factor	mean	SD^a^	ICC^b^	CI^c^= 95%	P-value	SEM^d^	MDC^e^	MDC%	Result
Patient care burden	43.88	12.29	0.98	0.99 – 0.96	P<0.001	1.73	4.79	10.91	Ideal
Conflict	56.19	4.02	0.97	0.98 – 0.94	P<0.001	0.69	1.91	9.76	Ideal
Positive perception of situations	10.94	2.08	0.91	0.96- 0.82	P<0.001	0.62	1.71	15.63	Acceptable
Self-actualization	16.21	3.55	0.89	0.95- 0.78	P<0.001	1.17	3.24	19.98	Acceptable
Fear and concern	15.14	4.95	0.95	-0.97-0.90	P<0.001	1.10	3.04	20.07	Acceptable
Total	105.76	21.21	0.97	0.99-0.95	P<0.001	3.67	10.17	9.61	Ideal

a: Standard Deviation;

b: Interclass Correlation Coefficient;

c: Confidence Interval;

d: Standard Error of Measurement;

e: Minimal Detectable Changes

### *Convergent Validity*

Correlation Coefficient between Quality of Life Inventory for Family Caregivers of Hemodialysis Patients and SF-8 Instrument was 0.554, indicating a good convergent validity. ^13^

### *Responsiveness*

The results of ANOVA revealed a significant difference for the quality of life among the three groups (with adequate, moderate and inadequate income). The LSD test (Post hoc analysis) also indicated that the caregivers with low income significantly had a lower quality of life than those with adequate and moderate income. Meanwhile, the moderate group had the significantly lower quality of life as compared to those with adequate income (F=11.54, df=2, P<0.001).

### *Interpretability*

Based on the following formula, for calculating the MIC, the standard deviation of the variations between the test-retest should be multiplied by the average effect size of 0.5. ^21^

MIC=0.5×SD of the ΔScore

The MIC must be higher than the MDC. ^22^ Given that the standard deviation of the test-retest test score was 21.21, the value of 10.60 was obtained from the multiplication, which is higher than the MDC value (10.17).

The ceiling and floor effects for the whole inventory were zero, and they were below 20% for the subscales, which are acceptable. 

### *Feasibility*

A lengthy inventory was prevented by performing accurate factor analysis. The time to respond the inventory ranged between 15 and 20 minutes.

### *Scoring*

The higher an individual’s score is, the higher overall score indicates better quality of life. The options were scored as follows: Strongly disagree (1), Disagree (3), Neither disagree nor agree (3), Agree (4), and Strongly Agree (5). All items, except for items 22 to 28, were conversely scored. The actual score of each item was obtained from the multiplication of the item score by its weight. For assigning the weight of each item, the factor load of every single item was multiplied by the ratio of each factor variance to the total variance (45.71). Then, the weight of each item was obtained from the ratio of the secondary values obtained from this stage to the sum of the secondary values (555.69). Naturally, after converting the scores to the standard score, the closer an individual’s mean score was to 100, the higher his/her quality of life would be.

## DISCUSSION

This study aimed to design and psychometrically assess the quality of life of family caregivers with hemodialysis patients. The qualitative phase of the study led to the formation of three themes, namely post-traumatic growth, objective and subjective burden of chronic care. People experience positive growth in the face of hardship. Finding ‘something positive’ as a consequence of suffering and hardship has been a hallmark of many religions cultures. ^27^ Post-traumatic growth is not limited to post-traumatic stress disorder. Family caregivers experience post-traumatic growth. Post-traumatic growth is also associated with quality of life and its protective role. ^28^ Other themes of the present study were the objective and subjective burden of chronic care. The burden of care, which includes the burden of objective and mental care, is the psychological, physical, and social stress that results from caring for patients with special medical needs. ^29^ In this regard, hemodialysis is a long process that affects all aspects of a patient’s and his or her family’s life, including physiological, psychological, functional, social, well-being and lifestyle. With increasing the care needs of patients undergoing dialysis, their caregivers face increasing burden of care and decreasing quality of life. ^8 , 29^

The results of the quantitative phase suggested that there were 34 items and five dimensions (including patient care burden, conflict, positive perception of situations, self-actualization, fear and concern) to explain the quality of life of family caregivers with hemodialysis patients. The first factor of the current inventory consists of 15 items referring to the negative consequences of caring on the caregivers’ different individual aspects such as physical and psychological complaints, disruptions in occupation and daily life, decision constraints, and fear of the future. This factor was called “patient care burden.” This factor with the highest number of items and weights is considered as one of the critical dimensions of this tool. This factor is also the primary determinant of the caregivers’ quality of life. ^30^ Quality of life and caregiver burden are inversely related among family caregiver of dialysis recipients. ^31^ The results of a study on 98 family caregivers in Ghana showed that 78% of family caregivers experienced a high level of patient care burden. ^32^

The second factor, which consists of 6 items, refers to the caregivers’ conflict and their patient care-relevant emotions, continuity of patient care, and social challenges. This factor was called “Conflict.” The items belonging to this dimension indicated that the category of culture, as one of the most prominent features of a local inventory, has been included. Conflict occurs in the intrapersonal, interpersonal, intra-group and inter-group forms. The conflict for caregivers can include issues such as inadequate support from the caregivers, lack of cooperation and participation of other family members in patient care, and exclusive care expectations from caregivers, different views on how care is taken, and the conflict between the caregiver and the care recipient. These lead to reduced quality of life. ^1^ For family caregivers, family conflict leads to adverse psychological effects such as depression, anger, stress, anxiety, and disturbance. ^33^ Patient care expectations are also influenced by cultural contexts. For example, passing the caring responsibility on a specific gender (female), or the caregiver and care recipient’s affinity (older son and wife/husband) is influenced by the cultures and norms of society. ^34^

The other factor in this inventory addresses the caregiver’s positive perception towards the current situation and consists of 3 items called “Positive perception of situations.” Researchers have admitted that caregivers can have both positive and negative perceptions towards patient care. ^31 , 35 , 36^ Satisfaction or dissatisfaction with care is affected by various personal, social and cultural factors. ^36^ Such different mental assessments make all people have a different understanding of situations despite the stressful context of care. In other words, some caregivers improve the psychological outcomes of care for themselves through holding a positive perception of the situations (good feeling of their situations or high self-esteem); however, some others feel stress more than it exists. ^27 , 31^ That brings negative consequences for the caregivers’ health and well-being, leading to the caregivers’ lower quality of life.

The fourth factor with four items refers to the potential and positive effects of patient care that is called self-actualization. Post-traumatic growth is a positive change experienced as a result of adversity. ^27^ Post-traumatic growth is associated with quality of life, and its existence is protective to caregivers. ^28^ Individual growth in family caregivers of patients undergoing hemodialysis disrupts the negative effects of care and helps the caregivers to obtain a flexible sense of care and support throughout this long and challenging time. ^37^

The fifth factor with six items covers the caregivers’ mental concern in care and is called “Fear and concern”. Long-term care, psychological problems, vital signs fluctuations, diet and drug observance, post-hemodialysis bleeding, and others may occur for hemodialysis patients, which, in turn, affect the caregivers. ^38^ Most caregivers often experience symptoms such as difficulty concentrating and remembering due to mental fatigue. ^39^

In this section, we compare the themes of the qualitative phase with the dimensions derived from the quantitative phase. Although the number of factors or dimensions of the present inventory is greater than the number of themes resulting from the qualitative phase, they are not mutually exclusive. The first dimension, the care burden of the same name, as one of the themes of the qualitative phase, is considered to be one of the most important dimensions of the inventory with the highest number of items and weight. Items belonging to the dimension of fear and concern also indicate that this dimension is consistent with the caring stressor subcategory resulting from the qualitative phase of the study. The items belonging to the conflict dimension are also consistent with the cultural and emotional burden subccategory that resulted from the qualitative phase of the study. Another dimension of this inventory is the positive perception of situations, which is consistent with the subcategory of positive caring beliefs of the self-efficacy category that resulted from the qualitative phase of the study. The self-actualization factor dimension is consistent with the theme of post-traumatic growth.

## CONCLUSION

The first step in attaining a fundamental knowledge of the hemodialysis patient caregiver’s life quality is access to a valid and reliable inventory which is consistent with the country’s socio-cultural context, so this inventory can be used in Iran’s care system. This inventory can be used in hospitals, clinics, outpatient clinics, and the community to assess the family caregivers’ quality of life and the consequences of the offered services. In research based on health policy, the quality of life scale can provide the decision makers and managers of the health system with useful information on the allocation of resources. 

One of the limitations of the present study was that it was not possible for the researcher to conduct interviews with family caregivers at their homes. Since confirmatory factor analysis was not performed, it is suggested that the construct validity of this inventory should be checked with confirmatory factor analysis.
